# Temple Syndrome: Clinical Findings, Body Composition and Cognition in 15 Patients

**DOI:** 10.3390/jcm11216289

**Published:** 2022-10-25

**Authors:** Alicia F. Juriaans, Gerthe F. Kerkhof, Eva F. Mahabier, Theo C. J. Sas, Nitash Zwaveling-Soonawala, Robbert N. H. Touwslager, Joost Rotteveel, Anita C. S. Hokken-Koelega

**Affiliations:** 1 Dutch Reference Center for Prader–Willi Syndrome/Prader–Willi-like, 3015 CN Rotterdam, The Netherlands; 2 Department of Pediatrics, Subdivision of Endocrinology, Erasmus MC/Sophia Children’s Hospital, 3015 CN Rotterdam, The Netherlands; 3 Dutch Growth Research Foundation, Westzeedijk 106, 3016 AH Rotterdam, The Netherlands; 4 Diabeter—Center for Pediatric and Adult Diabetes Care and Research, 3011 TA Rotterdam, The Netherlands; 5 Department of Pediatric Endocrinology, Emma Children’s Hospital, Amsterdam University Medical Centers, University of Amsterdam, 1105 AZ Amsterdam, The Netherlands; 6 Department of Pediatrics, Subdivision of Endocrinology, Wilhelmina Children’s Hospital, Utrecht University Medical Center, 3584 EA Utrecht, The Netherlands

**Keywords:** temple syndrome, maternal uniparental disomy 14, mUPD14, UPD14, Prader–Willi-like, Silver–Russell syndrome

## Abstract

Background: Temple syndrome (TS14) is an imprinting disorder caused by a maternal uniparental disomy of chromosome 14 (UPD(14)mat), paternal deletion of 14q32 or an isolated methylation defect of the MEG3-DMR. Studies on phenotypical characteristics in TS14 are scarce and patients with TS14 often experience delay in diagnosis, which has adverse effects on their health. TS14 is often characterized as either Prader–Willi-like, Silver–Russell-like or as a Silver–Russell spectrum disorder. Methods: This study describes 15 patients with TS14 who visited the Dutch Reference Center for Prader–Willi-like from December 2018 to January 2022. Results: Eight patients had UPD(14)mat and seven a methylation defect. The most common symptoms were intra-uterine growth retardation (IUGR) (100%), hypotonia (100%), precocious puberty (89%), small for gestational age (SGA) birth (67%), tube feeding after birth (53%) and psycho-behavioral problems (53%). Median (interquartile range (IQR)) IQ was 91.5 (84.25; 100.0), whilst many patients were enrolled in special education (54%). The median (IQR) fat mass % (FM%) SDS was 2.53 (2.26; 2.90) and lean body mass (LBM) SDS −2.03 (−3.22; −1.28). There were no significant differences in clinical characteristics between patients with a UPD(14)mat and a methylation defect. Conclusions: Our patients share a distinct phenotype consisting of IUGR, SGA birth, precocious puberty, hypotonia, tube feeding after birth, psycho-behavioral problems and abnormal body composition with a high FM% and low LBM. Whilst similarities with Prader–Willi syndrome (PWS) and Silver–Russell syndrome (SRS) exist, TS14 is a discernible syndrome, deserving a tailored clinical approach. Testing for TS14 should be considered in patients with a PWS or SRS phenotype in infancy if PWS/SRS testing is negative.

## 1. Introduction

Temple syndrome (TS14) is a rare condition caused by the abnormal expression of genes in the imprinted 14q32 region. The first patient with TS14 was described by Temple et al. in 1991 [1]. This case report presented a male with short stature, hydrocephalus, scoliosis, nasal speech, bifid uvula and small testes and he was diagnosed with maternal uniparental disomy of chromosome 14 (UPD(14)mat) with an underlying Robertsonian translocation inherited from his mother. 

TS14 can arise from several molecular causes, namely UPD(14)mat, with and without a Robertsonian translocation, paternal deletion and an isolated methylation defect [2,3,4,5]. All causes affect the 14q32.2 imprinted region, which is characterized by three differentially methylated regions (DMRs) and a cluster of paternally and maternally expressed genes [6]. The phenotype is caused by disruption of this region with loss of expression of the paternally inherited genes and overexpression of the maternally inherited genes [7]. It is not yet determined if the different etiologies cause an exactly identical phenotype or if there might be differences, though previous studies have alluded to the former [8].

The exact incidence and prevalence of TS14 are unknown, but there are fewer than 100 cases described in the literature. There are similarities with Prader–Willi syndrome (PWS) and Silver–Russell syndrome (SRS). TS14 is, therefore, associated with both a Prader–Willi-like phenotype and a Silver–Russell-like phenotype, showing symptoms that vary between patients, but also within the same patient from infancy into childhood and adulthood [9]. Patients with TS14 have been identified in cohorts of patients who tested negative for PWS and SRS [5,10,11,12,13]; thus, TS14 is included in the differential diagnosis of these syndromes [14,15]. 

The syndrome has not been extensively studied, but it has been shown that a subset of patients develops (truncal) obesity and there are reports of patients developing early-onset type 2 diabetes mellitus [16,17,18]. There is limited knowledge about cognitive development in patients with TS14, but it appears to range from normal to moderately delayed, although measured IQ is only reported in a few papers [18,19,20]. 

In this paper, we describe 15 patients with TS14. We present the patients’ body composition and cognitive functioning, and provide a comparison between the clinical characteristics of patients with a UPD(14)mat and an isolated methylation defect of the 14q32.2 region. We hypothesized that there would be no difference in clinical presentation between the etiologies. Furthermore, we report which symptoms are the most distinctive and common for TS14, analyze the patients’ journey to a diagnosis, and present a diagnostic flowchart based on the current study. Finally, we compare the phenotype of TS14 to that of PWS and SRS and propose a new conceptual approach to patients with TS14.

## 2. Patients and Methods

### 2.1. Design and Study Population

We carried out a prospective observational study in children and adolescents diagnosed with TS14 who visited our Dutch Reference Center for Prader–Willi syndrome/Prader–Willi-like between December 2018 and January 2022. Medical and family history of the patients were collected through a structured interview and assessed by the same examiner (A.F.J.). In some patients, the information was supplemented with data from medical records. Special education was defined as schools outside of the mainstream education with the aim to provide accommodated education for children with any type of disability. Psycho-behavioral problems were defined as any behavioral or psychiatric issues, which had an impact on the child, the family as a whole or the environment of the child. Hyperphagia was defined as insatiability, food-seeking behavior or food hoarding, and an increased focus on food. Both the psycho-behavioral problems and hyperphagia were assessed through a structured interview and observation by the same examiner (A.F.J.). The interview was performed in patients above the age of 7 years. In patients younger than 7 years, the parents were interviewed. The following are example questions, which were used to establish the presence of hyperphagia: are you/is your child always hungry? Are you/is your child always or often thinking about (the availability of) food? Do you/does your child feel hungry after eating a full meal? Do you/does your child wake up hungry at night and go looking for food? 

### 2.2. Anthropometric Measurements and Body Composition 

Standing height was measured with a Harpenden Stadiometer (Holtain, Ltd., Crosswell, UK). Weight was assessed on a calibrated scale (Servo Balance KA-20-150S; Servo Berkel Prior, Katwijk, The Netherlands). Height, weight and body mass index (BMI) SDS were calculated with Growth Analyser 4.0 (available at www.growthanalyser.org accessed on 4 February 2022), and were adjusted for gender and age according to Dutch reference values [21,22]. Small for gestational age (SGA) was defined as a birth weight below −2 standard deviations (SDS) [23]. 

In all children, fat mass % (FM%) and lean body mass (LBM) were measured with Dual-Energy X-ray Absorptiometry (DXA) (Lunar Prodigy; GE Healthcare, Chicago, IL, USA). All scans were conducted on the same machine, with daily quality assurance. The intra-assay coefficients of variation were 0.41–0.88% for fat tissue and 1.57–4.49% for LBM [24]. LBM was calculated as fat-free mass minus bone mineral content. FM was also expressed as percentage of total body weight (FM%). Standard deviation scores of FM, FM% and LBM were calculated according to age- and sex-matched Dutch Reference values [25]. 

### 2.3. Cognition 

Cognitive tests were performed by one experienced psychologist at the Reference Center for PWS/PWL, except in one patient, who had an intelligence test performed elsewhere. Wechsler Preschool and Primary Scale of Intelligence, Dutch Version (WPSSI-NL), was used for children below age 6 years. Wechsler Intelligence Scale for children (WISC) versions III and V were used in children between 6 and 16 years of age. WISC-V does not calculate a verbal and performance IQ, but has five indexes. Based on the revisions of indexes and subtests from WISC-III to WISC-IV and WISC-V [26,27,28,29], we used for this paper the verbal comprehension index as an approximation of the verbal IQ and in order to approximate the performance IQ, we averaged the visual spatial, fluid reasoning and processing speed indexes. The Wechsler Adult Intelligence Scale (WAIS) was used for adolescents above 16 years of age. In one patient, cognitive functioning was not formally tested, due to the anxiety it caused this patient. 

### 2.4. Statistical Analysis

Statistical analyses were performed using IBM SPSS statistics 25. Categorical variables are reported using frequencies and percentages. Continuous variables are expressed as median +/− interquartile range. Mann–Whitney U test was used to test the differences between genetic subtypes. 

### 2.5. Ethical Consideration

Ethical review and approval of this study were waived by the Medical Ethics Committee of Erasmus University Medical Center, Rotterdam, the Netherlands (MEC-2022-0065), because all data were collected as part of the clinical care. The study was conducted in accordance with the Declaration of Helsinki and the ethical standards of Erasmus University Medical Center. Written consent for publication was obtained from the patients and their caregivers.

## 3. Results

Clinical data are summarized in Table 1. A per patient overview is available in the Supplementary Table S1.

### 3.1. Genetic Diagnosis 

Eight patients were diagnosed with a UPD(14)mat (53%) and seven patients had an isolated methylation defect (47%) of the 14q32.2 locus. Prior to diagnosis and referral to our PWL Reference Center, many patients had an elaborate genetic diagnostic journey, including screening for metabolic diseases, karyotyping, CGH (comparative genomic hybridization) and SNP (single nucleotide polymorphism) array, several gene panels, WES (whole exome sequencing) panels, and PWS and SRS methylation tests. The initial genetic work-up was often done in infancy or early childhood, but testing for TS14 frequently only occurred years later, after the early onset of puberty. The median (IQR) age at diagnosis was 3.50 (2.04; 7.00) years. Only three patients were diagnosed before the age of two years. Patients with a UPD(14)mat had a lower age at diagnosis (2.55 (1.45; 4.63) years) compared to patients with a methylation defect (6.94 (2.46; 9.75) years), but this difference was not significant. 

### 3.2. Pregnancy and Delivery

One patient was conceived through in vitro fertilization (IVF). All patients had intra-uterine growth retardation (IUGR), diagnosed by serial prenatal ultrasounds. Other reported complications during pregnancy included: reduced fetal movements, low umbilical cord blood flow and hydronephrosis. The median (IQR) gestational age was 39.6 (37.1; 40.0) weeks. Only one patient was born premature at 34 weeks gestation. In four patients, labor was induced around 37 weeks gestation due to IUGR. A hypoplastic placenta was reported in one patient. Median (IQR) birth weight SDS was −2.51 (−3.10 to −1.40) and 67% was born SGA.

### 3.3. Growth and Maturation

All patients had neonatal hypotonia and 53% required tube feeding after birth for several days up to a month. At the time of examination, median (IQR) height SDS was −0.63 (−1.88 to 0.19) and four out of fifteen patients were receiving growth hormone (GH) treatment at that time. Two additional patients had received GH treatment until reaching adult height (AH). Median (IQR) height SDS without GH treatment in all patients was −1.80 (−2.95; −0.22). Height SDS without GH treatment was lower in children with a UPD(14)mat than in those with an isolated methylation defect (−2.33 vs. −0.24), but this difference was not significant. Without GH treatment, 7 out of 15 patients had short stature (47%). At examination, median (IQR) age was 9.02 (4.71; 12.47) and bone age was 9.00 (5.17; 14.25) years, while height SDS corrected for bone age was −1.42 (−2.54; 0.79), being lower than the height SDS not corrected for bone age, but this difference was not significant. Median weight for height SDS was 1.40 (−0.76 to 1.72) and median BMI SDS 1.45 (−0.73 to 1.68). Though weight for height SDS and BMI SDS did not differ significantly between patients with a UPD(14)mat and an isolated methylation defect, more patients with a methylation defect were overweight (43%) compared to patients with a UPD(14)mat (25%). All female patients (6/6; 100%) and two out of three male patients (67%) above age seven years had precocious puberty. All patients had small hands and feet.

### 3.4. Development and Behavior 

Median (IQR) IQ of the group was 91.50 (84.25; 100.0) and none of the patients had intellectual disability (ID) (IQ < 70). Many patients had a disharmonic intelligence profile, with a higher verbal IQ compared to the performance IQ. Median (IQR) verbal IQ was 98.50 (94.00; 108.75) and median (IQR) performance IQ was 89 (82.25; 97.25). Median (IQR) IQ was lower in patients with a UPD(14)mat (87.00 (79.00; 95.00)), compared to patients with methylation defect (98.00 (87.00; 109.00)), but this difference was not significant. Despite the fact that all patients had a total IQ above 70 and the median IQ was 92, 54% were enrolled in special education. Half of the patients had psycho-behavioral problems: 63% of patients with a UPD(14)mat and 43% of patients with an isolated methylation defect. These problems included autistic behavior (40%), of which 2 patients (33%) had a formal diagnosis of an autism spectrum disorder, obsessive and compulsive behavior (33%), aggressive behavior (20%), emotional outbursts (48%) and depression (20%). The psycho-behavioral problems had a negative impact on school performance. Additionally, 47% had hyperphagia. One patient with a methylation defect required constant supervision due to extreme hyperphagia, which included eating frozen foods and eating out of trash bins.

### 3.5. Body Composition

Median (IQR) FM% SDS was 2.53 (2.26; 2.90) and median (IQR) LBM SDS was −2.03 (−3.22 to −1.28). Eight patients had an LBM < −2 SDS (53%) and 13 patients had an FM% >2 SDS (87%). The lowest LBM SDS was present in the youngest patients, whereas the older patients had a higher FM% SDS. LBM SDS was lower in patients with UPD(14)mat (−2.12 (−3.08; −1.32)) compared to patients with a methylation defect (−1.75 (−3.63 to −1.28)), but this difference was not significant. 

### 3.6. Other Findings 

There were some facial characteristics that were noted in most patients, such as a broad and high forehead, frontal bossing, a small mouth and a triangular face. Half of the patients had a simian crease, most often observed on the left hand. Two patients had a bifid uvula and one a cleft palate; 40% of patients had recurrent otitis media and two patients had anosmia. Joint hypermobility was found in 67% of patients. Two patients presented with episodes of fainting, which was evaluated extensively, but no apparent cause was found. Many patients had dental problems, including severely misaligned teeth and dental crowding. A few patients had a history of cystic lesions, such as benign brain cysts, a thyroglossal cyst and a choledochal cyst. One patient had dilated cardiomyopathy and a bicuspid aortic valve. In this patient, the diagnosis of UPD(14)mat was a coincidental finding in a WES, carried out to find the underlying cause for the cardiomyopathy. Another patient was diagnosed with late-onset congenital adrenal hyperplasia (CAH), which came to light soon after she was diagnosed with TS14. 

## 4. Discussion 

This study describes the clinical features of 15 patients with TS14, including 7 with an isolated methylation defect. In addition, we measured body composition and cognitive functioning. The most common symptoms were prenatal growth failure (100%), neonatal hypotonia (100%), small hands and/or feet (100%), joint hypermobility (67%) and precocious puberty (89% of patients above age 7 years). Of 15 patients, 7 had a simian crease (47%), 8 (53%) required tube feeding after birth and 67% were born small for gestational age (birth weight SDS ≤ −2). Seven patients (47%) had hyperphagia. 

To our knowledge, this is the first study with body composition measurements by DXA scan in a large group of children with TS14. We found a high median (IQR) FM% SDS of 2.53 (2.26; 2.90) and a low median LBM SDS of −2.03 (−3.22 to −1.28). The overall abnormal median body composition was also present in the patients who were treated with GH. Strikingly, even though median FM% was >2 SDS, median BMI SDS was 1.45 and therefore within the normal range. Thus, body composition analysis is more helpful in detecting TS14 and during follow-up than is calculating BMI. This is comparable to the body composition profile with low LBM and high FM% of patients with PWS [30]. 

The median (IQR) IQ of the patients in the current study was 95 (86; 102), which is in line with a previous report [8], but in a different study, intellectual disability was more common [9]. None of the patients were intellectually disabled, based on the IQ score. The patient with the lowest full scale IQ of 76 had, in addition to a UPD(14)mat, a partial trisomy 14, which could have negatively affected her intellectual development. Despite the fact that there were no patients with intellectual disability, 54% (7/13) was enrolled in special education. This was mostly due to social-emotional developmental delay and psycho-behavioral problems, such as autism and emotional outbursts, which negatively impacted school performance. Many patients had a disharmonic intelligence profile, with a much higher verbal IQ compared to the performance IQ, which might have led to an increased likelihood of an inability to keep up in mainstream education.

There were some differences in features and symptoms in patients with a UPD(14)mat compared to patients with an isolated methylation defect, but none were statistically significant. As TS14 is very rare, our sample size was small, which led to a limited statistical power. Nevertheless, we would like to comment on some noticeable trends. Patients with a methylation defect were diagnosed later in childhood than patients with a UPD(14)mat (6.94 vs. 2.55 years of age). Another study also found patients with a methylation defect were on average diagnosed at a later age [8]. This is perhaps because a UPD(14)mat can be preferentially found through SNP array, which is often a first-choice diagnostic test, or it can be detected in the genetic work-up of patients with a Robertsonian translocation. Isolated methylation defects, however, can only be found by methylation analysis of the 14q32 locus. Furthermore, although patients with a UPD(14)mat and those with an isolated methylation defect had abnormal body composition, with an even higher FM% and lower LBM in patients with a UPD(14)mat, overweight/obesity was more common in patients with a methylation defect (43% vs. 25%). The median IQ of patients with an isolated methylation defect was higher (98 vs. 87) and they had fewer psycho-behavioral problems (43%) compared to patients with a UPD(14)mat (63%). Tube feeding after birth was more common in patients with a UPD(14)mat than in those with a methylation defect (75 vs. 29%), as was joint hypermobility (75% vs 57%) and recurrent otitis media (50% vs. 29%).

Due to the fact that the different etiologies that cause TS14 result in the same methylation levels in the 14q32.2 region [2], differences between the UPD(14)mat and the methylation defect group might be caused by other factors. Patients with an isodisomy, with homozygous regions as a consequence, could have manifestations of recessive disorders, which can also give rise to symptoms not native to the syndrome [31]. Another explanation might be that patients with TS14 could have additional erroneous methylated regions in other areas of the genome, causing symptoms unrelated to TS14 [5,32,33]. These imprinting disorders on regions other than 14q32.2 could also be an explanation for the wide spectrum of phenotypical expression between patients with TS14, and the fact that some patients have similarities with patients with PWS and others with SRS [7,34].

Our results are more or less in line with a review of published cases [9], which found that most patients were born small for gestational age (SGA) (87%) had speech (59%) and motor delay (83%), and an early onset of puberty (86%). Other symptoms included relative macrocephaly, feeding difficulties, congenital abnormalities and intellectual disability. However, in that review, short stature was very common (79%), whereas in our population, short stature without GH treatment was less prevalent (47%). In the current study, four patients were receiving GH treatment at the time of the study, and two had received GH until reaching adult height. As GH treatment has been proven to improve growth [35], this could possibly explain that an average height SDS was found at the time of examination. Several patients in our study had precocious puberty and had gone through their pubertal growth spurt at a young age and most had been treated with puberty-inhibiting GnRH (Gonadotropin Releasing Hormone) analogues. As a consequence, their current height SDS does not accurately predict their final height and some of our patients might qualify as having short stature in adulthood. The study by Ioannides et al. also includes adult patients and possibly some patients were never treated with GnRH analogues. This may have caused the higher percentage of patients with short stature. Several of our patients had a higher bone age compared to their chronological age and their height SDS corrected for bone age was indeed lower than their height SDS (−1.42 vs. −0.63), indicating that adult height will be much lower than expected based on the current height SDS. This difference was more striking in patients with a methylation defect (−2.31 vs. −0.24).

Patients with TS14 have been described as both Prader–Willi-like [8,9,15,36,37] and Silver–Russell-like and as a Silver–Russell spectrum disorder [8,9,12,13,38]. Some characteristics are similar to both SRS and PWS and seem to be age-dependent [8,9], such as the SRS facial appearance with a high and prominent forehead and relative macrocephaly, which seems to become less apparent with age [8], and the prevalence of overweight, which is more common in adolescents with TS14 [9]. Many patients in our group had a Prader–Willi-like phenotype (i.e., hypotonia, hyperphagia, overweight/obesity), but not all. The neonatal phenotype of TS14 was quite similar to that of PWS, but our patients with TS14 had a lower birth weight than is generally seen in patients with PWS [39]. The body composition profile of patients with TS14 with a high FM% and low LBM is more similar to that of patients with PWS [30] than to that of those with SRS [40]. Two patients in our study, however, met 4 out of the 6 criteria of the Netchine–Harbison clinical scoring system (NH-CSS) at the age of 24 months and would thus qualify for a clinical diagnosis of SRS [11,41]. However, our patients had a normal to high FM%, which is very rare in young children with SRS [40]. Unlike in PWS and SRS, a large number of patients did not require tube feeding after birth (47%) and those with tube feeding required it for days instead of several weeks to months as in PWS [39] or even years as can occur in SRS [11]. Other distinctive characteristics of TS14, such as precocious puberty, a disharmonic intelligence profile and specific behaviors, are uncommon or completely lacking in PWS and SRS. Such unique aspects of TS14, combined with the overall phenotype, require a specific treatment plan tailored to the syndrome. With regard to precocious puberty, this should include anticipatory action and alertness in order to identify precocious puberty and start GnRH treatment as early as possible. It is important to monitor bone age regularly by radiological assessment because the advanced bone age of patients even years prior to the early start of puberty serves as a warning that precocious puberty is imminent. The advanced bone age also has a negative impact on adult height and the efficacy of GH treatment. Patients with TS14 often have an average IQ, but because of the discrepancy between verbal and performance IQ, they often require special education. Furthermore, the higher verbal IQ often results in overestimation of their abilities. It is important that attention is given to their special needs in terms of education and guidance. Advice and guidance on hyperphagia requires a different approach in children with TS14 compared to those with PWS due to the fact that patients with TS14 are often more independent in daily life. This gives them more opportunities to find food outside of the controlled home environment. Lastly, the specific psycho-behavioral problems that people with TS14 might have, such as obsessive–compulsive tendencies, emotional outbursts and autism spectrum disorder, are similar to those of patients with PWS, but may require a different approach which corresponds better to their cognitive ability. Some patients with TS14 also struggle with acceptance of their chronic disorder and develop depression. By continuing to consider TS14 to be either a Prader–Willi-like or Silver–Russell-like syndrome, a custom designed approach to the treatment of this syndrome will not be developed. Even though there are similarities between the syndromes, we would like to advocate considering TS14 a discernible syndrome in its own right, different from both PWS and SRS.

**Figure 1:**
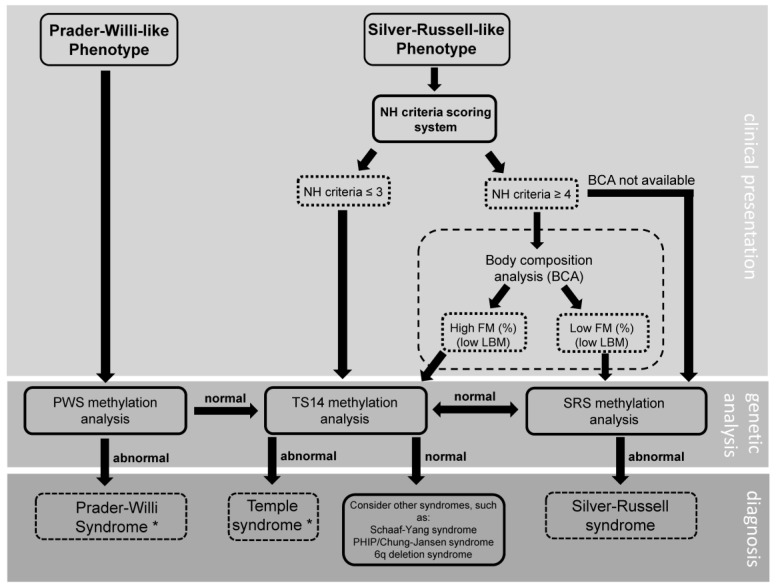
Clinical diagnostic flowchart for patients with a Prader–Willi-like or Silver–Russell-like phenotype. NH: Netchine–Harbison; FM: fat mass, LBM: lean body mass, PWS: Prader–Willi syndrome; TS14: Temple syndrome; SRS: Silver–Russell syndrome; BCA: body composition analysis. * after establishment of diagnosis, further testing to identify the genetic subtype should follow.

Many of the patients in the current study had a long diagnostic odyssey before finally arriving at their diagnosis. As previously mentioned, this was especially true for patients with a methylation defect. Often, a TS14 diagnosis would only be considered after the onset of early puberty. This not only negatively impacted the children and their caregivers mentally, but also had a negative effect on their health. Up until the moment of diagnosis, suitable multidisciplinary care and treatment was not available to them. This caused a delay in adequate weight management, timely intervention in behavior and appropriate handling of early puberty and/or persistent short stature, which could result in undesirable outcomes. To streamline the diagnostic process and avoid delay, we provide a flowchart based on our study (Figure 1), with a proposed approach to the patient with either a Prader–Willi-like or a Silver–Russell-like phenotype. In this flowchart, we recommend testing for TS14 in patients with a Prader–Willi-like phenotype and a negative PWS methylation test. In addition, we suggest using the Netchine–Harbison Clinical Scoring System (NH-CSS) and a body composition analysis, if readily available, as a means of discerning between patients with SRS and those with TS14. Different from the international consensus for diagnosis and management of patients with SRS [42], in our diagnostic approach, one would test a patient with an SRS-like phenotype and an NH-CSS of ≤3, for TS14. Furthermore, body composition analysis is not a part of the diagnostic route in the international consensus, but when it is easily available, it could be a good option to distinguish between SRS and TS14.

Our study details one of the larger cohorts of patients with TS14, with also a significant number of patients with an isolated methylation defect, allowing us to explore the potential differences between the subgroups. Another strength of our study is the fact that we were able to collect data in patients with TS14, such as body composition, which was not previously reported in a group this large and in such detail. Furthermore, we give a comprehensive overview of the, often elaborate, diagnostic journey of the patients.

The patients in the current study were all referred to the Dutch Reference Center for Prader–Willi-like (PWL) patients. Theoretically, this could have caused a bias in the presented phenotypes, as all patients who were referred would in theory always present with a PWL phenotype. However, most patients were referred after our national call for patients with an existing TS14 diagnosis, independent of the presenting phenotype. Some patients were already being treated with GH and/or GnRH analogues, which presented a challenge when it came to assessing growth and stature, but we do also provide the height before and without GH therapy. Ideally, to further map out the TS14 phenotype, one would study a group of untreated patients with TS14. However, withholding treatment from patients with TS14 is considered unethical and therefore not feasible.

Future research in patients with TS14 should focus on the efficacy of the treatment options, such as treatment with GH to increase height SDS and improve body composition. Moreover, the psycho-behavioral problems and developmental delay should be assessed more systematically, preferably with a DSM classification. It would also be interesting to have a more detailed study on the correlation between the need for special education and the intelligence profiles and/or specific psycho-behavioral problems. Finally, it would be valuable to explore the differences between the genetic subgroups in larger groups of patients with TS14.

In conclusion, we present 15 patients with TS14, sharing a distinct phenotype of IUGR, SGA birth, hypotonia, tube feeding after birth, hyperphagia, precocious puberty, psycho-behavioral problems and abnormal body composition with a high FM% and low LBM. There were no significant differences between patients with a UPD(14)mat and an isolated methylation defect. Whilst most patients had an IQ in the normal range, half of the patients were enrolled in special education. The neonatal phenotype of TS14 is very similar to that of PWS and some patients might qualify for a clinical diagnosis of SRS according to the Netchine–Harbison criteria. Whilst similarities between the syndromes exist, TS14 is a multi-faceted, discernible syndrome, deserving a tailored clinical approach. In order to avoid delay in diagnosis, we advocate testing for TS14 if suspicion of PWS or SRS remains after a negative test or in patients born with SGA with precocious puberty with unknown cause in later childhood. Body composition analysis can aid in distinguishing between the syndromes.

## Figures and Tables

**Table 1:** Summary of clinical manifestations of patients with TS14.

	UPD(14)mat	Methylation Defect	Total
Number	8	7	15
Sex (male:female)	5:3	3:4	8:7
Age at examination (y)	10.76 (5.32; 15.64)	7.00 (2.76; 9.99)	9.02 (4.71; 12.47)
Age of diagnosis (y)	2.55 (1.45; 4.63)	6.94 (2.46; 9.75)	3.50 (2.04; 7.00)
Perinatal			
Gestational age	39.8 (37.48; 40.00)	37.60 (37.00; 40.10)	39.6 (37.10; 40.00)
Premature delivery	1/8 (13%)	0/7 (0%)	1/15 (7%)
Prenatal growth failure	**8/8 (100%)**	**7/7 (100%)**	**15/15 (100%)**
Birth weight-SDS	−2.84 (−3.38 to −2.36)	−1.53 (−2.64 to −0.91)	−2.51 (−3.10 to −1.40)
SGA	**7/8 (88%)**	3/7 (43%)	**10/15 (67%)**
Growth			
Short stature (before GH treatment)	**4/8 (50%)**	3/7 (43%)	7/15 (47%)
(history of) GH treatment	5/8 (63%)	1/7 (14%)	6/15 (40%)
Height-SDS before GH treatment	−2.06 (−3.24; −1.22)	**−0.26 (−2.64; 0.19)**	−1.80 (−2.95; −0.22)
Height-SDS at examination	−1.05 (−1.79 to 0.18)	−0.26 (−2.54; 0.19)	−0.63 (−1.88 to 0.19)
Bone age at examination	10.65 (5.35; 15.41)	8.80 (2.80; 12.00)	9.00 (5.17; 14.25)
Height-SDS corrected for bone age	−1.03 (−1.38; −0.29)	−2.07 (−2.72; −1.42)	−1.42 (−2.54; −0.79)
BMI			
BMI-SDS before GH treatment	1.24 (−1.06; 2.11)	1.50 (−2.28; 2.25)	1.45 (−1.17; 2.18)
BMI SDS at examination	1.34 (−0.28; 2.17)	1.45 (−2.28 to 1.54)	1.45 (−0.73 to 1.68)
Puberty			
Precocious puberty *	**4/5 (80%)**	**4/4 (100%)**	**8/9 (89%)**
(History of) GnRH treatment	4/8	2/7	6/15
Duration of GnRH-treatment (months)	48.00 (44.25; 63.00)	43.50 ^†^	46.00 (43.00; 53.00)
Developmental status			
Total IQ	87.00 (79.00; 95.00)	98.00 (87.00; 109.00)	91.50 (84.25; 100.00)
Verbal IQ	98.00 (85.00; 100.00)	106.00 (94.00; 115.00)	98.50 (94.00; 108.75)
Performance IQ/visual-spatial	87.00 (80.00; 94.00)	95.00 (83.00; 98.00)	89.00 (82.25; 97.25)
Intellectual disability (IQ < 70)	0/8 (0%)	0/7 (0%)	0/15 (0%)
Special education	**4/8 (50%)**	**3/5 (60%)**	**7/13 (54%)**
Psycho-behavioral problems	**5/8 (63%)**	3/7 (43%)	**8/15 (53%)**
Body composition			
FM%	44.10 (35.55; 50.38)	39.70 (33.80; 44.40)	41.10 (34.30; 48.50)
FM% SDS	2.56 (2.32; 2.88)	2.54 (0.96; 2.95)	2.53 (2.26; 2.90)
LBM SDS	−2.12 (−3.08; −1.32)	−1.75 (−3.63 to −1.28)	−2.03 (−3.22 to −1.28)
Other findings			
Hypotonia	**8/8 (100%)**	**7/7 (100%)**	**15/15 (100%)**
Small hands and/or feet	**8/8 (100%)**	**7/7 (100%)**	**15/15 (100%)**
Simian crease	**4/8 (50%)**	3/7 (43%)	7/15 (47%)
Joint hypermobility	**6/8 (75%)**	**4/7 (57%)**	**10/15 (67%)**
Scoliosis	3/8 (38%)	2/7 (29%)	5/15 (33%)
Tube feeding after birth	**6/8 (75%)**	2/7 (29%)	**8/15 (53%)**
Overweight/obesity	2/8 (25%)	3/7 (43%)	5/15 (33%)
Hyperphagia	**4/8 (50%)**	3/7 (43%)	7/15 (47%)
Recurrent otitis media	**4/8 (50%)**	2/7 (29%)	6/15 (40%)
Anosmia	0/8 (0%)	2/7 (29%)	2/15 (13%)
Bifid uvula/cleft palate	1/8 (13%)	2/7 (29%)	3/15 (20%)
SRS according to NH-CSS	1/8 (13%)	1/7 (14%)	2/15

Data are shown as the median (interquartile range) or frequency (numerator/denominator). Bold: 50% and above. No significant differences between UPD(14)mat and methylation defect subgroup. Short stature was defined as height <−2 SDS. Height SDS before GH treatment includes height of all patients, including untreated patients. SGA: small for gestational age; GH: growth hormone; BMI: body mass index; IQ: intelligence quotient; GnRH: gonadotropin releasing hormone; LBM: lean body mass; FM: fat mass; * precocious puberty applicable to patients above 7 years of age; ^†^ only 2 patients, no IQR provided.

## Data Availability

The datasets generated during and/or analyzed during the current study are not publicly available but are available from the corresponding author on reasonable request.

## Supplementary Materials

The following supporting information can be downloaded at: https://www.mdpi.com/article/10.3390/jcm11216289/s1, Table S1: Clinical data of the individual patients with TS14.
