# Supplementary material for: Temple Syndrome: Clinical Findings, Body Composition and Cognition in 15 Patients
Source: J Clin Med. 2022 Oct 25;11(21):6289. doi: 10.3390/jcm11216289 (PMC9656486; doi:10.3390/jcm11216289)
Supplement: Supplementary file 1 [file jcm-11-06289-s001.zip › jcm-1922366-supplementary.pdf]

Supplementary Table S1: Clinical data of the individual patients with TS14

	Patient 1	Patient 2	Patient 3	Patient 4	Patient 5	Patient 6
Sex	Female	Male	Male	Male	Male	Male
Age at examination (y)	1.8	2.8	4.2	4.7	5.3	5.4
Genetic subtype	Methylation defect	Methylation defect	UPD-14	Methylation defect	UPD-14	UPD-14
Age of diagnosis	1 year 9 months	2 years 6 months	1 year 3 months	4 years 5 months	2 years 1 month	2 years 11 months
Genetic diagnostic history	-Metabolic screen<br>-SNP-array<br>-WES panel ID<br>-multilocus methylation test	-Metabolic screen<br>-WES panel ID<br>-WES panel small stature<br>-CFTR-gene<br>-Multilocus methylation test	-SNP-array<br>-Trio SNP analysis	-Array CGH<br>-Obesity panel<br>-WES panel obesity<br>-PWS methylation<br>-TS14 methylation	-Metabolic screen<br>-SNP-array<br>-Gene panel cardiomyopathy<br>-WES trio analysis	Unknown
Perinatal period						
Gestational age	40	37	37	41	41	38
Premature delivery	-	-	-	-	-	-
Prenatal growth failure	+	+	+	+	+	+
Birth weight-SDS	-2.65	-1.53	-2.73	-3.10	-2.94	-2.37
Growth						
GH treatment at examination	-	-	-	-	-	-
Height-SDS before GH treatment	NA	NA	NA	NA	NA	NA
Height-SDS at examination	-2.54	-2.64	-3.13	0.48	-1.34	0.34
Bone age at examination	1.80	2.80	3.90	6.00	5.17	5.90
Height SDS corrected for bone-age at examination	-2.54	-2.72	-2.67	-1.42	-1.17	-0.39
BMI						
BMI-SDS before GH treatment	NA	NA	NA	NA	NA	NA
BMI SDS at examination	-4.34	-2.74	-1.42	2.05	-0.73	4.68
Puberty						
Precocious puberty*	NA	NA	NA	NA	NA	NA
(History of) GnRH treatment	-	-	-	-	-	-
Duration of GnRH treatment (months)	NA	NA	NA	NA	NA	NA
Developmental status						
IQ (TIQ)	87	111	85	95	79	87
Verbal IQ	94	115	100	94	78	98
Performance IQ	83	111	80	98	89	85

Intellectual disability (TIQ < 70)	-	-	-	-	-	-
Special education	NA	NA	-	-	-	+
Psycho-behavioral problems	-	-	+	-	+	-
Body composition						
FM%	33.20%	33.80%	39.30%	39.70%	33.00%	52.00%
FM% SDS	0.14	0.96	2.52	2.54	2.18	3.05
Lean body mass SDS	-4.32	-3.63	-3.51	-1.33	-2.64	-1.15
Clinical diagnosis						
Prader-Willi-like phenotype	-	-	-	-	-	+
Netchine-Harbison criteria score	4	3	4	0	2	0
Other findings						
Hypotonia	+	+	+	+	+	+
Small hands and/or feet	+	+	+	+	+	+
Simian crease	-	+	-	+	+	+
Joint hypermobility	-	-	+	+	+	+
Scoliosis	+	-	-	-	+	-
Tube feeding after birth	-	-	+	+	-	+
Duration of tube feeding (days)	NA	NA	5	4	NA	unknown
Overweight/obesity	-	-	-	+	-	+
Hyperphagia	-	-	-	+	-	+
Recurrent otitis media	-	-	-	-	-	+
Anosmia	-	-	-	-	-	-
Bifid uvula/cleft palate	-	-	-	-	-	+
Facial characteristics	Almond shaped eyes, high and prominent forehead, frontal bossing, small nose and mouth	Triangular face, prominent forehead, small mouth	Frontal bossing, short philtrum, small mouth	-	High and prominent forehead, hypertelorism, small mouth	-
Additional features		Dental problems	Hydronephrosis, dental problems		Cardiomyopathy, bicuspid aortic valve, choledochal cyst	

	**Patient 7**	**Patient 8**	**Patient 9**	**Patient 10**	**Patient 11**	**Patient 12**
Sex (male:female)	Female	Female	Male	Female	Male	Female
Age at examination (y)	7	9.02	9.05	9.99	11.72	12.47
Genetic subtype	Methylation defect	Methylation defect	UPD-14	Methylation defect	Methylation defect	UPD-14
Age of diagnosis	7 years	7 years 4 months	3 years 6 months	9 years 9 months	11 years and 4 months	2 years
Genetic diagnostic history	-CGH array<br>-PWS	-karyotype<br>-PWS	-CGH array<br>-PWS	-SNP array<br>-Gene panel	- PWS methylation	-Metabolic screen

	methylation<br>-metabolic screen<br>-WES<br>-SRS methylation<br>-TS14 methylation	methylation<br>-TS14 methylation	methylation	obesity<br>-WES<br>-multilocus methylation	-SRS methylation<br>- SNP array<br>- TS14 methylation	-sub telomeric MLPA<br>- SNP array
Perinatal						
Gestational age	37	37	40	40	37.6	40
Premature delivery	-	-	-	-	-	-
Prenatal growth failure	+	+	+	+	+	+
Birth weight-SDS	-0.41	-1.40	-3.95	-2.51	-0.91	-2.35
Growth						
GH-treatment at examination	-	+	+	-	-	+
Height-SDS before GH treatment†	NA	-2.95	-2.33	NA	NA	-1.80
Height-SDS at examination	-0.26	-0.63	-0.30	0.19	-0.22	0.58
Bone age at examination	8.80	9.20	9.00	12.00	16.40	12.30
Height-SDS corrected for bone age at examination	-2.07	-0.79	-0.26	-1.58	-3.91	0.73
BMI						
BMI-SDS before GH treatment	NA	2.25	1.18	NA	NA	1.30
BMI-SDS at examination	1.98	1.35	1.44	3.61	1.45	1.84
Puberty						
Precocious puberty (>7 years)	+	+	-	+	+	+
(History of) GnRH treatment	-	+	-	+	-	+
Duration of GnRH treatment (months)	NA	44	NA	43	NA	48
Developmental status						
Total IQ	98	82	unknown	98	109	95
Verbal IQ	108	99	unknown	106	130	97
Performance IQ	97	68	unknown	89	95	94
Intellectual disability (IQ<70)	-	-	-	-	-	-
Special education	+	+	+	+	-	+
Psycho-behavioral problems	++	++	+	-	+	-
Body composition						
FM%	43.50%	44.40%	41.10%	50.80%	36.50%	48.50%
FM% SDS	2.95	2.86	2.6	3.25	2.38	2.90
Lean body mass (SDS)	-1.75	-2.36	-2.22	0.16	-1.28	-1.80
Clinical diagnosis						

Prader-Willi-like phenotype	+	+	-	+	-	-
Netchine-Harbison criteria score	1	2	unknown	2	1	2
Other findings						
Hypotonia	+	+	+	+	+	+
Small hands and/or feet	+	+	+	+	+	+
Simian crease	-	+	-	-	-	-
Joint hypermobility	+	+	-	+	-	-
Scoliosis	-	-	-	+	-	+
Tube-feeding after birth	+	-	+	-	-	-
Duration of tube-feeding (days)	unknown	NA	unknown	NA	NA	NA
Overweight/obesity	+	-	-	+	-	-
Hyperphagia	+	+	+	-	-	-
Recurrent otitis media	-	+	+	+	-	-
Anosmia	+	-	-	+	-	-
Bifid uvula/cleft palate	+	-	-	+	-	-
Facial characteristics	Flat facies, high forehead, small nose	Flat facies, high and prominent forehead	Broad forehead, deep-set nasal bridge, small nose	-	Broad forehead	Almond shaped eyes, upslant of the eyes, small mouth, retrognathia, broad forehead, smooth philtrum
Additional features	Late-onset CAH, Hypoplastic placenta, extreme fatigue, dental problems, (benign) cystic brain lesions, thyroglossal duct cyst	Unexplained syncope, extreme fatigue, dental problems, conductive hearing loss, chronic respiratory tract infection	Cryptorchidism, dental problems	Unexplained syncope, (benign) cystic brain lesion	Tube feeding after age 8 months, atopy, ASS	Dental problems

	**Patient 13**	**Patient 14**	**Patient 15**
Sex (male:female)	Male	Female	Female
Age at examination (y)	15.27	15.76	16.08
Genetic subtype	UPD-14	UPD-14	UPD-14 + partial trisomy
Age of diagnosis	7 years 2 months	5 years	9 months
Genetic diagnostic history	-Array CGH<br>-SRS methylation<br>-SNP array<br>-TS14 methylation	-Karyotype<br>-FISH<br>-PWS methylation<br>-FMR1 gene<br>-MLPA for CNVs<br>-Subtelomeric MLPA<br>-markers chromosome 14	-Array CGH
Perinatal			
Gestational age	34	40	39
Premature delivery	+	-	-

Prenatal growth failure	+	+	+
Birth weight-SDS	-1.18	-3.19	-3.44
Growth			
GH-treatment at examination	+	-‡	-‡
Height-SDS before GH treatment	-3.27	-1.18	-3.36
Height-SDS at examination	-1.88	-0.76	-1.50
Bone age at examination	14.25	17.50	15.80
Height SDS corrected for bone-age	-1.10	-0.96	-1.45
BMI			
BMI-SDS before GH treatment	-1.53	1.93	2.18
BMI SDS at examination	1.34	2.73	1.98
Puberty			
Precocious puberty*	+	+	+
(History of) GnRH treatment	+	+	+
Duration of GnRH treatment (months)	68	48	43
Developmental status			
Total IQ	88	106	76
Verbal IQ	98	111	85
Performance IQ	87	99	72
Intellectual disability (IQ<70)	-	-	-
Special education	-	-	+
Psycho-behavioral problems	-	+	+
Body composition			
FM%	34.30%	51.00%	47.10%
FM% SDS	2.26	2.82	2.52
Lean body mass (SDS)	-3,22	-1.16	-2.02
Clinical diagnosis			
Prader-Willi-like phenotype	-	+	-
Netchine-Harbison criteria score	unknown	3	3
Other findings			
Hypotonia	+	+	+
Small hands and/or feet	+	+	+
Simian crease	+	-	+
Joint hypermobility	+	+	+
Scoliosis	+	-	-

Tube-feeding after birth	+	+	+
Duration of tube-feeding (days)	30	30	unknown
Overweight/obesity	-	+	-
Hyperphagia	-	+	+
Recurrent otitis media	+	+	-
Anosmia	-	-	-
Bifid uvula/cleft palate	-	-	-
Facial characteristics	Triangular face, small mouth, broad forehead, large ears	Prognathism, high forehead, low set ears	-
Additional features		ASS, leg length discrepancy, extreme fatigue, dental problems	ASS, Growth hormone deficiency, central hypothyroidism

Note: NA: not applicable; SGA: small for gestational age; GH: growth hormone; BMI: body mass index; IQ: intelligence quotient; GnRH: gonadotropin releasing hormone; LBM: lean body mass; FM: fat mass; ASS: autism spectrum disorder * precocious puberty applicable to patients above 7 years of age; † not applicable to patients who are not treated with GH, for height SDS before treatment, also see height SDS at examination; ‡GH treatment stopped after reaching adult height.
